# Instruments

**Published:** 1891-02

**Authors:** 


### Instruments.

Prof. Woodbury, of the Medico-Chirurgical College of Philadelphia, commences a paper in the follow language:

" It is a trite observation that every workman requires good tools in order to do his best work. If this work is inferior in quality the fact that he used poor tools will not excuse him to his patrons.

He is held responsible for, and is judged by his results, and failure will be attributed to lack of ability, or practical skill.

It is, therefore, the duty of every master workman to see that his tools are of good quality, and that they are in perfect order."

In a like manner it is the duty of every dentist to personally acquaint himself with the quality and character of every instrument he uses, and also of the action of every drug that comes within his province to use. We are so apt to become empiricists! Let each and every action or thought be that of reason. Gain a knowledge that will serve you at all times. This knowledge must be *practical* to succeed as a dentist. Theory has its place, and it helps to enlarge our studious natures.

What is the dental profession ?

It is a collective body of persons engaged in the practice of preserving the natural teeth and contiguous parts, also supplying artificial means in event of the loss of any part, or otherwise adding to the organs such conditions as may be deemed necessary for use and beauty.

What is the prime object of our work?

Naturally we work for a subsistence, but when a given operation is to be performed, the object that the patient seeks is *result!* We strive for a perfect result of our work, and then the pecuniary reward follows in exchange for the result the patient has porcured through our skill and knowledge. We must, then, to produce these results have the means at our command, and this implies *good* instruments, *good* remedies, *good* eyes, *good* skill, *good every thing!*

Dentists have become lax in their selection of instruments; selecting perhaps the cheaper ones. If, then, we can not pay a fair price for a *good* instrument is it any wonder then that the market is flooded with instruments of poor material and ill-tempered?

The remedy for the evil is to demand of the manufacturers a superior article, and pay them for it. It is cheaper to buy one first-class instrument than several poor ones which will last only as long as the good instruments.

This is an important matter, and it is golden advice to the beginner when told to select fewer instruments, and those of the very best material. These can be had at any time of reliable firms. Quantity of instruments do not make the dentist. Of course there are a large number of instruments that are necessary. The maintenance of proper care of instruments we hope to speak of in a future number of the Register. W.

